# Dipeptidyl peptidase-4 inhibitors reduced long-term cardiovascular risk in diabetic patients after percutaneous coronary intervention via insulin-like growth factor-1 axis

**DOI:** 10.1038/s41598-022-09059-2

**Published:** 2022-03-24

**Authors:** Yuichi Chikata, Hiroshi Iwata, Katsutoshi Miyosawa, Takuma Koike, Hidetoshi Yasuda, Takehiro Funamizu, Shinichiro Doi, Hirohisa Endo, Hideki Wada, Ryo Naito, Manabu Ogita, Tomotaka Dohi, Takatoshi Kasai, Kikuo Isoda, Shinya Okazaki, Katsumi Miyauchi, Tohru Minamino

**Affiliations:** 1 Department of Cardiovascular Biology and Medicine, Juntendo University Graduate School of Medicine, 2-1-1 Hongo, Bunkyo-ku, Tokyo, Japan; 2 Tokyo New Drug Research Laboratories, Kowa Company, Ltd., Tokyo, Japan; 3 Department of Cardiology, Juntendo University Nerima Hospital, Tokyo, Japan; 4 Department of Cardiology, Juntendo University Shizuoka Hospital, Shizuoka, Japan

**Keywords:** Biomarkers, Cardiology

## Abstract

Dipeptidyl-peptidase-4 inhibitors (DPP4i) have been the most used antidiabetic medications worldwide due to their good safety profiles and tolerability with a low risk of hypoglycemia, however, large cardiovascular outcome trials (CVOTs) have not shown any significant the prognostic superiority. On the contrary, since observational studies have suggested the effects of DPP4i are enhanced some populations, such as Asians and those who without overweight, their prognostic benefit is still under debate. The aim of this study was thus to assess the prognostic impact of DPP4i in patients with both diabetes and coronary artery disease (CAD) who underwent percutaneous coronary intervention (PCI) through the insulin-like growth factor-1 (IGF-1) axis, a substrate of DPP4. This single-center analysis involved consecutive Japanese diabetic patients who underwent PCI for the first time between 2008 and 2018 (n = 885). Primary and secondary endpoints were set as cardiovascular (CV) death and the composite of CV death, non-fatal myocardial infarction and ischemic stroke (3P-MACE). Serum levels of IGF-1 and its main binding protein (insulin-like growth factor binding protein-3: IGFBP-3) were measured. In consequences, unadjusted Kaplan–Meier analyses revealed reduced incidences of CV-death and 3P-MACE by DPP4i, which was particularly enhanced in patients who were not overweight (BMI ≤ 25). Multivariate Cox hazard analyses consistently indicated reduced risks of CV death by DPP4i at PCI (hazard ratio (HR) 0.39, 95% confidence interval (CI) 0.16–0.82, p = 0.01) and 3P-MACE (HR 0.47, 95% CI 0.25–0.84, p = 0.01), respectively. Moreover, elevated IGF-1 activity indicated by the IGF-1/IGFBP-3 ratio was associated with decreased risks of both endpoints and it was significantly higher in patients with DPP4i (p < 0.0001). In conclusion, the findings of the present study indicate beneficial effects of DPP4i to improve outcomes in Japanese diabetic patients following PCI, which might be mediated by DPP4–IGF-1 axis.

## Introduction

Diabetes mellitus, a firmly established risk for atherosclerotic cardiovascular (CV) mortality and morbidity, such as coronary, cerebral and peripheral artery disease, is a major socioeconomic burden worldwide^1,2^. Appropriate glycemic control in combination with multifactorial risk control is thus essential in order to avoid macrovascular complications that can lead to critical consequences in diabetic patients. Among multiple lines of anti-diabetic medications, dipeptidyl peptidase-4 inhibitors (DPP4i) are a major antidiabetic agent class in the United States^3^, European Union^4,5^ and Japan^6^. Although accumulated experimental evidence has suggested an atheroprotective effects of DPP4 inhibition^7–9^, previous major large-scale cardiovascular outcome trials (CVOTs) of DPP4i which primarily aimed to establish their non-inferiority compared to conventional anti-diabetic therapy, have not demonstrated a clear benefit with respect to improving outcomes in populations which mainly consisted of diabetic individuals with a history of CV disease (secondary prevention)^10–13^. In contrast, 2 observational studies have reported differences in the efficacy of DPP4i among populations, including the superiority of the glucose lowering effect of DPP4i in Asians and normal weight compared to obese individuals^14,15^. Moreover, a study using a Korean national database showed the substantial prognostic merit of DPP4i at reducing CV risk, and the composite of all-cause death, stroke and myocardial infarction in a population without a history of CV disease^16^. Accordingly, the differences regarding the prognostic impact of DPP4i among subgroups remain obscure and inconclusive.

In this study, we thus investigated the prognostic efficacy of DPP4i in Japanese patients with diabetes following percutaneous coronary intervention (PCI) by using a prospective single-center PCI registry database. Moreover, to elucidate the potential target molecules of DPP4 and the benefits of its inhibition, we measured the serum levels of insulin-like growth factor-1 (IGF-1), as one of the substrates for catalytic degradation by DPP4 and its regulatory protein, insulin-like growth factor binding protein-3 (IGFBP-3), and assessed their prognostic effects in association with the use of DPP4i.

## Results

### Baseline characteristics in patients with or without DPP4i

The background demographics, comorbidities and medications are listed and compared between groups with and without DPP4i in Table 1. Most of the background demographic parameters, including age, sex, BMI, and diabetes duration were similar, while the proportion of ACS was significantly higher in the DPP4i (−) group. Parameters regarding target lesions and number of diseased vessels were not different between the two groups. Total cholesterol, low-density lipoprotein-cholesterol (LDL-C) and glycated hemoglobin (HbA1c-NG) levels were lower in patients with DPP4i compared to those without. With respect to conventional antidiabetic medications, the prevalence of insulin use was almost doubled in the DPP4i (−) group compared to the DPP4i (+) group, while that of metformin was higher in the DPP4i (+) group. Despite a low prevalence, the use of sodium–glucose cotransporter 2 (SGLT2) inhibitors was higher in the DPP4i (+) group. The ratios of statins and ezetimibe use were higher in the DPP4i (+) group in concert with lower LDL-C. Moreover, comparisons of baseline characteristics between patients with low BMI (≤ 25) or not in DPP4i (+) and (−) groups found that patients with low BMI were older and not likely to have dyslipidemia and there was a significant deference in the concomitant use of SGLT-2 inhibitor with DPP4i between groups with BMI > 25 and ≤ 25 (Supplementary Table 1).

**Table 1:** Baseline characteristics of study patients.

	Overall	DPP4i (+)	DPP4i (−)	p-value
	n = 885	n = 324	n = 561	
**Baseline characteristics**				
Age, years	68.0 ± 10.1	68.1 ± 10.0	67.9 ± 10.2	0.84
Male, n (%)	733 (82.8)	269 (83.0)	464 (82.7)	0.90
Body mass index, kg/m^2^	24.7 ± 3.8	24.8 ± 4.0	24.7 ± 3.7	0.50
Hypertension, n (%)	694 (78.4)	247 (76.2)	447 (79.7)	0.23
Dyslipidemia, n (%)	691 (78.1)	254 (78.4)	437 (77.9)	0.86
Current smoker, n (%)	207 (23.4)	72 (22.2)	135 (24.1)	0.53
Chronic kidney disease, n (%)	272 (30.7)	97 (29.9)	175 (31.2)	0.70
Hemodialysis, n (%)	75 (8.5)	23 (7.1)	52 (9.3)	0.26
Acute coronary syndrome, n (%)	214 (24.2)	62 (19.1)	152 (27.1)	**0.008**
LVEF, %	60.6 ± 12.7	60.2 ± 12.7	60.7 ± 12.7	0.65
Diabetes duration, years	14 (6, 22)	13 (8, 20)	14 (5, 22)	0.92
Number of diseased vessels	2.0 ± 0.8	1.9 ± 0.8	2.0 ± 0.8	0.12
**Vessel location**				
RCA, n (%)	268 (30.3)	91 (28.1)	177 (31.6)	0.28
LAD, n (%)	449 (50.7)	176 (54.3)	273 (48.7)	0.10
LCX, n (%)	169 (19.1)	63 (19.4)	106 (18.9)	0.84
**Laboratory findings**				
TC, mg/dL	166.5 ± 38.5	160.9 ± 33.2	169.7 ± 40.9	**0.001**
LDL-C, mg/dL (Friedewald)	96.2 ± 31.7	92.7 ± 27.3	98.2 ± 33.8	**0.01**
HDL-C, mg/dL	43.3 ± 13.3	42.6 ± 12.5	43.8 ± 13.8	0.20
TG, mg/dL	119 (86, 163)	120 (85, 160)	119 (87, 163)	0.63
FBG, mg/dL	134.9 ± 56.1	132.9 ± 53.1	136.1 ± 57.8	0.42
HbA1c-NG, %	7.2 ± 1.1	7.1 ± 1.0	7.3 ± 1.1	**0.02**
hs-CRP, mg/L	0.09 (0.03, 0.29)	0.08 (0.03, 0.30)	0.09 (0.03, 0.28)	0.61
Hemoglobin, g/dL	13.3 ± 1.9	13.3 ± 1.8	13.3 ± 2.0	0.55
eGFR, mL/min/1.73 m^2^	69.1 ± 28.5	71.2 ± 29.7	67.9 ± 27.8	0.10
1,5-AG, μg/mL	8.6 (4.1, 14.4)	8.7 (4.1, 14.7)	8.6 (4.1, 14.3)	0.69
BNP, pg/mL	50.4 (23.0, 133.6)	42.5 (19.4, 111.8)	57.9 (24.1, 148.4)	**0.04**
AIP	0.46 ± 0.29	0.46 ± 0.28	0.46 ± 0.29	0.93
**Medication**				
Sulfonylurea, n (%)	234 (26.4)	99 (30.6)	135 (24.1)	**0.03**
Metformin, n (%)	170 (19.2)	77 (23.8)	93 (16.6)	**0.009**
Thiazolidinedione, n (%)	94 (10.6)	30 (9.3)	64 (11.4)	0.32
SGLT-2 inhibitor, n (%)	17 (1.9)	12 (3.7)	5 (0.9)	**0.003**
GLP-1 receptor agonist, n (%)	6 (0.7)	1 (0.3)	5 (0.9)	0.31
α-Glucosidase inhibitor, n (%)	190 (21.5)	62 (19.1)	128 (22.8)	0.20
Glinide, n (%)	72 (8.1)	38 (11.7)	34 (6.1)	**0.003**
Insulin, n (%)	223 (25.2)	48 (14.8)	175 (31.2)	**< 0.0001**
ACE-I/ ARB, n (%)	485 (54.8)	172 (53.1)	313 (55.8)	0.44
β-Blocker, n (%)	405 (45.8)	149 (46.0)	256 (45.6)	0.92
Statin, n (%)	598 (67.6)	242 (74.7)	356 (63.6)	**0.001**
Ezetimibe, n (%)	51 (5.8)	26 (8.0)	25 (4.5)	**0.03**
Fibrate, n (%)	34 (3.8)	11 (3.4)	23 (4.1)	0.60

*LVEF* left ventricular ejection fraction, *RCA* right coronary artery, *LAD* left anterior descending artery, *LCX* left circumflex artery, *TC* total cholesterol, *LDL-C* low-density lipoprotein, *HDL-C* high-density lipoprotein, *TG* triglycerides, *FBG* fasting blood glucose, *HbA1c-NG* glycated hemoglobin, *hs-CRP* high-sensitivity C-reactive protein, *eGFR* estimated glomerular filtration rate, *1,5-AG* 1,5-anhydroglucitol, *BNP* b-type natriuretic peptide, *AIP* atherogenic index of plasma (Log TG/HDL-C), *SGLT-2* sodium–glucose co-transporter-2, *GLP-1* glucagon-like peptide-1, *ACE-I* angiotensin-converting enzyme inhibitors, *ARB* angiotensin receptor blockers.

### Prognostic merit of DPP4i in patients after PCI

**Figure 1:**
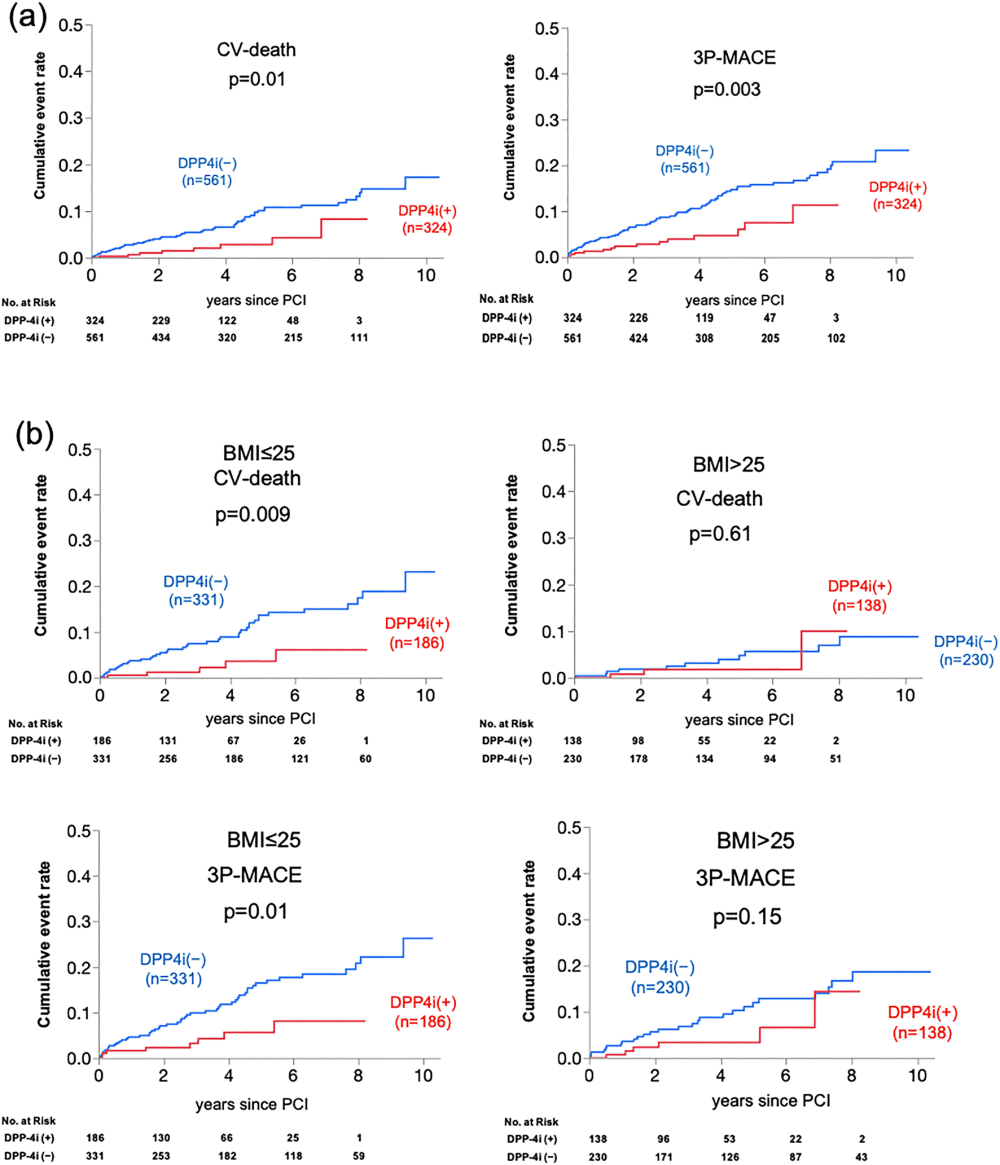
Cumulative incidences of adverse cardiovascular events following PCI in patients with and without DPP4i. (**a**) Cumulative incidences of CV death and 3P-MACE in patients treated with and without DPP4i (DPP4i (+) and (−) groups). (**b**) Cumulative incidences of CV death and 3P-MACE in DPP4i (+) and (−) groups in patients with and without low BMI (≤ and > median BMI, 25). *CV* cardiovascular, *3P-MACE* 3-point major adverse cardiovascular events, the composite of CV death, non-fatal myocardial infarction and ischemic stroke.

During the follow-up period after PCI, the numbers of identified CV deaths and 3P-MACE were 59 (6.7%) and 89 (10.1%) out of 885 participants, respectively. Unadjusted Kaplan–Meier analysis followed by the log-rank comparison test showed that the cumulative incidences of both primary and secondary endpoints were significantly lower in the DPP4i (+) group compared to the DPP4i (−) group indicating a possible favorable prognostic effect of DPP4i in this population following PCI (Fig. 1a). Of interest, such an impact of DPP4i was significant only in individuals who were not overweight (BMI ≤ 25), while the cumulative incidences of both CV death and 3P-MACE in the DPP4i (+) and (−) groups were similar amongst overweight patients and obese patients (Fig. 1b).

**Figure 2:**
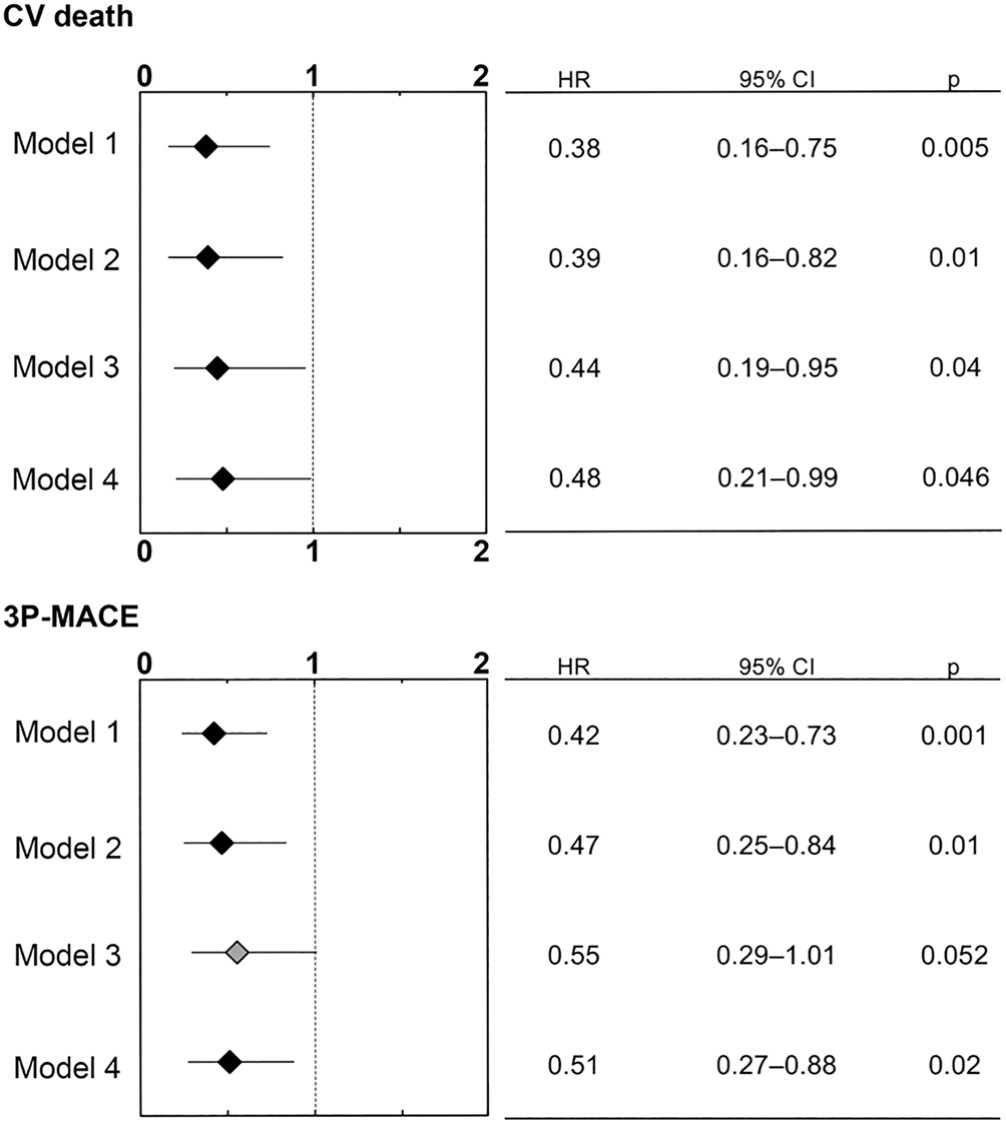
Prognostic impact of DPP4i at PCI for adverse cardiovascular events in 4 Cox proportional hazard models. Hazard ratios were calculated by multivariate Cox proportional hazard analyses by using 4 different models. Model 1: age, sex and DPP4i, Model 2: age, sex, body mass index (BMI), chronic kidney disease (CKD), b-type natriuretic peptide (BNP), insulin and DPP4i, Model 3: age, sex, acute coronary syndrome (ACS), left ventricular ejection fraction (LVEF), statins, hemoglobin and DPP4i, Model 4: metformin, sulfonylurea, alpha-glucosidase inhibitors, thiazolidinedione, glinide, insulin and DPP4i. *HR* hazard ratio, *95% CI* confidence interval.

Univariate Cox proportional hazard analyses for calculating hazard ratios for subsequent CV death and 3P-MACE after PCI revealed that DPP4i at procedure was significantly associated with reduced risk of both endpoints (Supplementary Tables 2 and 3). Multivariate Cox proportional analyses using 3 models (Models 1–3) in which covariates were selected based on background demographics and univariate analyses (Supplementary Tables 2 and 3) showed that DPP4i at procedure significantly lowered the risks of CV death and 3P-MACE (Fig. 2). Furthermore, multivariate analyses using another model (Model 4), which included diabetic medications, such as metformin, sulfonylurea, alpha-glucosidase inhibitors, thiazolidinedione, glinide, and insulin in addition to DPP4i indicated that only DPP4i was associated with lower risk of both endpoints (Fig. 2).

### Beneficial effect of DPP4i exerted potentially through IGF-1 axis

**Figure 3:**
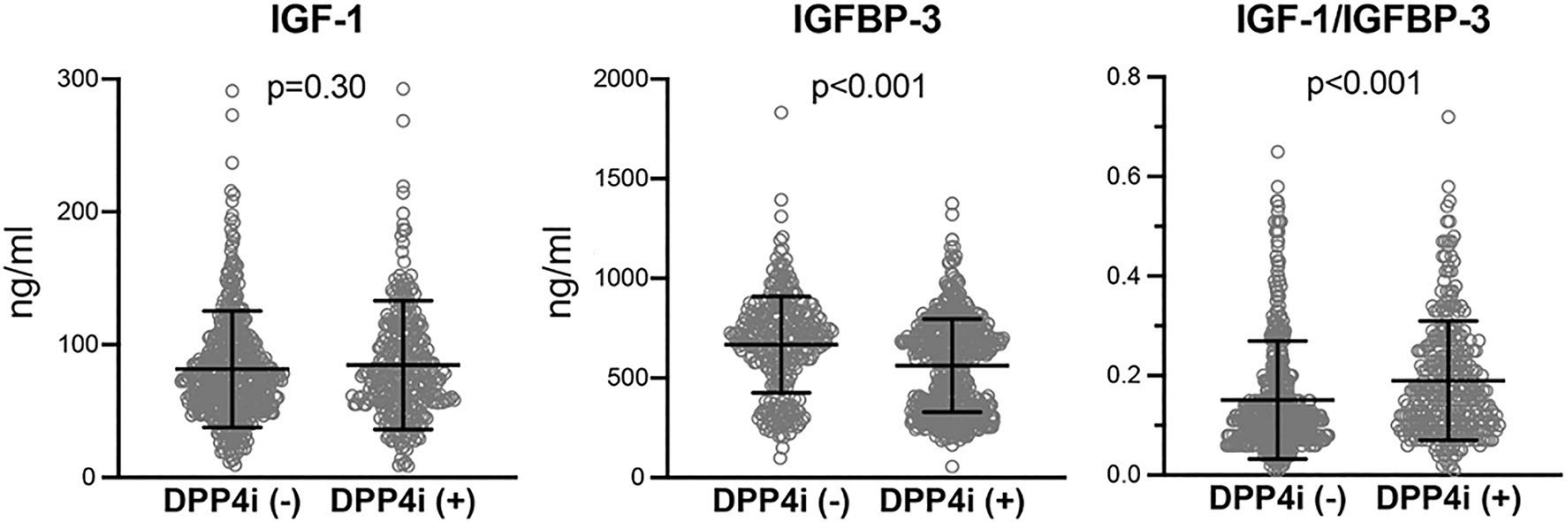
Comparison of serum levels of IGF-1, IGFBP-3 and IGF-1/IGFBP-3 ratio in diabetic patients with and without DPP4i. Serum levels of IGF-1, IGFBP-3 and IGF-1/IGFBP-3 ratio. Each open circle represents one participant. The horizontal lines indicate average ± standard deviation, respectively.

In an attempt to elucidate a portion of the potential mechanisms which mediate the favorable prognostic impact of DPP4i in the present population, we measured the serum concentrations of IGF-1, one of the major substrates of DPP4^17^, and its regulatory molecule IGFBP-3 in the entire population. As shown in Fig. 3, despite no significant difference in the serum levels of IGF-1 in the two groups (76.3 ng/mL [57.3, 104.3] vs. 73.9 ng/mL [54.7, 98.2], p = 0.30), IGFBP-3 levels were significantly attenuated (519.3 ± 218.1 ng/mL vs. 650.8 ± 241.7 ng/mL, p < 0.0001) and the ratio of IGF-1/IGFBP-3, which is a known indicator of IGF-1 activity^18^, was significantly amplified in the DPP4i (+) group compared to the DPP4i (−) group (0.16 [0.10, 0.25] vs. 0.12 [0.08, 0.18], p < 0.0001). Moreover, unadjusted Kaplan–Meier analyses when dividing the participants into two groups according to the median of IGF-1/IGFBP-3 revealed that the cumulative incidences of both CV death and 3P-MACE were constantly and significantly lower in patients with a high IGF-1/IGFBP-3 ratio (Supplementary Fig. 2a). Furthermore, differences in the cumulative incidences of both endpoints between the high and low IGF-1/IGFBP-3 groups were enhanced in patients who were not overweight (BMI ≤ 25) and attenuated in those who were overweight or obese (BMI > 25) (Supplementary Fig. 2b), findings that were substantially comparable with those in Kaplan–Meier analyses when the participants were divided based on the presence or absence of DPP4i (Fig. 1a,b). Moreover, multivariate analysis including age, sex, BMI, chronic kidney disease, BNP and insulin use (Model 2) revealed that an elevated IGF-1/IGFBP-3 ratio was associated with a significantly decreased risk for CV death and 3P-MACE (Supplementary Table 5).

## Discussion

This single-center observational study of a prospective PCI registry database involving consecutive 885 Japanese diabetic patients primarily demonstrated that the use of DPP4i at the time of PCI procedure was associated with avoiding subsequent CV death and 3P-MACE. The merit of DPP4i was considerably augmented in patients with a lower BMI. Moreover, the ratio of IGF-1 to IGFBP-3, one of the enzymatic substrates of DPP4 to its main transport protein indicating the bioavailability of IGF-1, was significantly higher in patients with DPP4i. Kaplan–Meier analyses, as well as unadjusted and adjusted Cox proportional hazard analyses showed that a high IGF-1/IGFBP-3 ratio was associated with a significantly lower risk of CV death and 3P-MACE after PCI. Moreover, very similar to analyses with and without DPP4i, the prognostic impact of the IGF-1/IGFBP-3 ratio was enhanced in patients with a lower BMI.

DPP4, a multifunctional circulating or cell surface protein, is present on a wide range of cell types and exerts a variety of biological activities, such as protease activity, interaction with the extracellular matrix, and regulation of intracellular signaling^17,19^. Inhibition of DPP4 lowers blood glucose by inhibiting the catalytic degradation of the incretin hormones glucagon-like peptide-1 (GLP-1) and glucose-dependent insulinotropic polypeptide (GIP), both of which promote insulin secretion from pancreatic beta-cells, and inhibit glucagon secretion and glucose production in the liver^20,21^. Other than incretins, there are physiological substrates of DPP4 that are bioactive molecules cleaved by DPP4, such as BNP, neuropeptide Y, stromal cell-derived factor-1α (SDF-1α) and IGF-1^17,22^. Multiple lines of previous experimental evidence have demonstrated the cardioprotective and anti-atherogenic effects of DPP4i^7–9^. However, landmark large-scale randomized CVOTs of DPP4i which mainly involved patients with a history of CV disease (secondary prevention) did not show any clear benefit for reducing the risk of the composite of CV events^10–13^. Although these studies had been primarily designed to identify the non-inferiority, and not the superiority, of DPP4i compared to conventional diabetic medications, the study results and their clinical impacts were considerably different to those of SGLT-2 inhibitors^23,24^ and GLP-1 receptor agonists^25,26^. However, observational studies have indicated that the effects of DPP4i might not be uniform among ethnicities or those with different BMI^14,15^. The Diabetes Epidemiology: Collaborative analysis Of Diagnostic criteria in Europe (the DECODE) and in Asia (the DECODA) studies from the World Health Organization (WHO) demonstrated that both insulin resistance and secretion capacity are higher in Caucasians than Asians^27–29^. In Western countries, insulin resistance, which frequently occurs in conjunction with obesity, is the predominant pathophysiological issue, while impaired insulin secretion is a major issue in Asians^30^ and a genetic difference in relation to diabetes between Asians and non-Asians has been also reported^31^. In addition to ethnicity, it has been reported BMI might alter the effects of DPP4i^14^. DPP4 enzymatic activity was suggested to be upregulated in proinflammatory situations, including obesity, and it was reported to be significantly positively correlated with BMI^19,32^.

Consistent with these previous findings regarding the possible superiority in the effects of DPP4i in Asians^30,31^ and individuals with low BMI^14,19,32^, DPP4i in the present study was found to be beneficial at reducing the risk of CV death and 3P-MACE after PCI in the entire study population. Moreover, the merit of DPP4i was substantially enhanced in patients with a lower BMI (≤ 25), although the proportion of SGLT-2 inhibitor use was significantly lower in these patients with DPP4i. The present findings indicate that there might be subpopulations, such as Asians and those without overweight, who specifically benefit from DPP4i, although large scale CVOTs involving a limited number of Asians (9–20%)^10–13^ and patients with low BMI failed to show any significant merit.

Among various substrates of DPP4, which are catalytically deactivated by DPP4, previous experimental studies have suggested a variety of cardioprotective effects of IGF-1, including the improvement of endothelial function, the enhancement of plaque stability, and the inhibition of vascular inflammation^33–35^. Consistently, an observational study indicated that low circulating IGF-1 and high IGFBP-3 levels were significantly associated with increasing the risk of developing ischemic heart disease in a primary prevention population^36^. In this study, serum IGF-1 levels were similar in both patients with and without DPP4i, but IGFBP-3 levels were significantly reduced and the IGF-1/IGFBP-3 ratio was significantly increased in patients with DPP4i. Since the IGF-1/IGFBP-3 ratio has been considered to be an indicator of IGF-1 bioavailability^18^, these findings suggest that administration of DPP4i might increase its bioavailability in this population, which in turn may lead to better outcomes. Consistently, prognostic analyses of the IGF-1/IGFBP-3 ratio by Kaplan–Meier and Cox proportional analyses have indicated its elevation was significantly associated with a reduced risk of adverse outcomes after PCI. Moreover, similar to that for DPP4i, the favorable prognostic impact of the IGF-1/IGFBP-3 ratio was substantially augmented in the population that was not overweight. Taking these findings together, the beneficial prognostic effects of DPP4i in this population may be exerted through the DPP4–IGF-1 axis.

There are several limitations to this study. First, it is single centered and retrospective and included only Japanese patients, and therefore did not compare findings amongst different ethnicities. The possibility that unknown confounders might have had an influence on the results could not be completely denied, even though multivariate analyses adjusted for baseline characteristics and known prognostic factors. Second, the number of patients treated with newer generations of antidiabetic medications, such as SGLT-2 inhibitors and GLP-1 receptor agonists, was quite small. This should have been taken into consideration as confounders of multivariate analyses. Third, this study did not assess heart failure hospitalization as an endpoint, although a trial revealed that a DPP4i, saxagliptin, was associated with a higher incidence of heart failure hospitalization compared to placebo^10^. Therefore, larger-scale and further updated future studies involving participants with newer classes of antidiabetic drugs should assess heart failure as an outcome as well.

In conclusion, this retrospective observational study demonstrated that patients treated with DPP4i and those with a higher IGF-1/IGFBP-3 ratio were associated with lower risks of CV mortality and 3P-MACE in Japanese diabetic patients after PCI, especially in lower BMI patients. These findings suggest that the benefit of DPP4i might be mediated by the DPP4–IGF-1 axis.

## Methods

### Ethical approval

This study followed the Declaration of Helsinki and was approved by the Institutional Review Board (IRB) of Juntendo University (IRB-ID: 20-287). The single-center prospective all-comer registry database of patients who underwent any type of PCI at Juntendo University Hospital, Tokyo, Japan (Juntendo Physicians’ Alliance for Clinical Trial, J-PACT) since 1984 is publicly registered (University Medical Information Network Japan—Clinical Trials Registry, UMIN-CTR 000035587). Written informed consent was obtained from all participants for the J-PACT registry which had no exclusion criteria as far as written informed consent was achieved^37,38^.

### Participants, endpoints, follow-up, and follow-up duration

This study is a retrospective analysis of a portion of the J-PACT registry database involving consecutive 885 diabetic patients out of 4039 entire patients who underwent any type of PCI for coronary artery disease (CAD) for the first time at Juntendo University Hospital, Tokyo, Japan between December 2008 and January 2018. Diabetes was defined as HbA1c ≥ 6.5% or with any diabetic medications at PCI procedure in this study. Participants were divided into two groups; with or without DPP4i (DPP4i (+) n = 324, DPP4i (−) n = 561) at PCI procedure, and the incidence and risk of subsequent endpoints following PCI were assessed (Supplementary Fig. 1).

The primary endpoint was set as CV-related death (CV death) and the secondary endpoint was the composite of CV death, non-fatal myocardial infarction and ischemic stroke (3-point major adverse cardiovascular events: 3P-MACE). CV death was defined as a composite of the following types of death; sudden death in which non-cardiac death could not be excluded, and death due to myocardial infarction, heart failure, cardiogenic shock, a cerebrovascular event, or aortic diseases. In this prospective PCI registry database, patient follow-up was based on chart review, as far as they were followed at Juntendo University Hospital. A prognosis survey questionnaire was mailed out every 5 years if they were transferred to other institutions. When there was no response to the questionnaire, further follow-up was conducted by phone call. In cases in which no response was achieved by either, follow-up was terminated at the latest time point, at which their survival at our institution was confirmed, such as the last visit date to an outpatient clinic or the last day of any hospitalization. The median and the range of the follow up period were 1456 and 0–3785 days, respectively.

### Blood sampling and the measurement of serum levels of IGF-1 and IGFBP-3

Blood samples were obtained immediately prior to PCI through an inserted blood access (5 to 8 French catheter) and stored at − 80 °C until measurement of IGF-1 and IGFBP-3 concentrations (n = 816). Serum levels of IGF-1 and IGFBP-3 were determined using commercially available enzyme-linked immunosorbent assay (ELISA) kits (Duoset DY291 and DY675; R&D Systems, USA) according to the manufacturer’s protocol. For Kaplan-Maier analyses, patients were divided into 2 groups by the median IGF-1/IGFBP-3 (= 0.126) as low and high IGF-1/IGFBP-3 ratio group, respectively.

### Statistical analysis

Quantitative variables are presented as the mean ± standard deviation or median with interquartile range (IQR) in accordance with the results of the Shapiro–Wilk normality test. Categorical variables are presented as the numbers and percentages. Quantitative data between groups were compared using the Student’s *t*-test or the Wilcoxon rank sum test. Unadjusted Kaplan–Meier analysis was used to evaluate the time to the cumulative incidence of endpoints followed by the log-rank test for comparisons. Univariate and multivariate analyses using the following models of Cox proportional hazards regression analyses (Model 1–4) were used to calculate the hazard ratios (HRs) of DPP4i and the IGF-1/IGFBP-3 ratio for endpoints. Variables used in Models 1–3 were selected based on the background demographics different between the groups and were associated with endpoints in univariate analyses. Model 1 included age (a continuous variable) and sex. Model 2 included age (a continuous variable), sex, body mass index (BMI) (a continuous variable), chronic kidney disease (≥ stage 3), b-type natriuretic peptide (BNP) (a continuous variable) and insulin. Model 3 included age (a continuous variable), sex, acute coronary syndrome (ACS), left ventricular ejection fraction (LVEF), statins, and hemoglobin. Additionally, Model 4 included other diabetic medications, including metformin, sulfonylurea, alpha-glucosidase inhibitors, thiazolidinedione, glinide and insulin. Statistical significance was defined as a p-value < 0.05 and analyses were performed using statistical software (JMP Pro 12.0; SAS Institute Inc., Cary, NC, USA and IBM SPSS Statistics, Version 26.0. Armonk, NY, USA).

### Ethics approval and consent to participate

This study was performed in accordance with the Declaration of Helsinki and was approved by the Institutional Review Board (IRB) of Juntendo University (IRB-ID: 20-287), and all patients gave their written informed consent to participate in the study.

## Supplementary Information

Supplementary Information.

## Data Availability

The datasets used and/or analyzed during the current study are available from the corresponding author on reasonable request.
