# Supplementary material for: Dipeptidyl peptidase-4 inhibitors reduced long-term cardiovascular risk in diabetic patients after percutaneous coronary intervention via insulin-like growth factor-1 axis
Source: Sci Rep. 2022 Mar 24;12:5129. doi: 10.1038/s41598-022-09059-2 (PMC8948237; doi:10.1038/s41598-022-09059-2)
Supplement: Supplementary file 1 — Supplementary Information. [file 41598_2022_9059_MOESM1_ESM.docx]

**SUPPLEMENTAL MATERIALS**

**Supplementary Table 1**

Background demographics of patients with BMI > 25 and ≤ 25 in DPP4i (+) and (-) groups

|  | DPP4i (+) | | | |  | DPP4i (-) | | |
| --- | --- | --- | --- | --- | --- | --- | --- | --- |
|  | **BMI>25** | **BMI≤25** | **P** | |  | **BMI>25** | **BMI≤25** | **P** |
|  | n=138 | n=186 |  |  |  | n=230 | n=331 |  |
| **Baseline characteristics** |  |  |  | |  |  |  |  |
| Age, years | **64.8 ± 10.0** | **70.5 ± 9.2** | **<0.0001** | |  | **65.1 ± 10.5** | **69.9 ± 9.5** | **<0.0001** |
| Male, n (%) | 117 (84.8) | 152 (81.7) | 0.47 | |  | 186 (80.9) | 278 (84.0) | 0.34 |
| Hypertension, n (%) | 112 (81.2) | 135 (72.6) | 0.07 | |  | 192 (83.5) | 255 (77.0) | 0.06 |
| Dyslipidemia, n (%) | **117 (84.8)** | **137 (73.7)** | **0.02** | |  | **192 (83.5)** | **245 (74.0)** | **0.008** |
| Current smoker, n (%) | 35 (25.4) | 37 (19.9) | 0.24 | |  | 51 (22.2) | 84 (25.4) | 0.76 |
| Chronic kidney disease, n (%) | 44 (31.9) | 53 (28.5) | 0.51 | |  | 67 (29.1) | 108 (32.6) | 0.77 |
| Hemodialysis, n (%) | 9 (6.5) | 14 (7.5) | 0.73 | |  | 20 (8.7) | 32 (9.7) | 0.15 |
| ACS, n (%) | 30 (21.7) | 32 (17.2) | 0.3 | |  | 66 (28.7) | 86 (26.0) | 0.48 |
| LVEF, % | 60.3 ± 12.9 | 60.2 ± 12.7 | 0.97 | |  | 62.0 ± 11.4 | 59.9 ± 13.4 | 0.12 |
| Diabetes duration, years | 15 (9, 22) | 13 (8, 20) | 0.93 | |  | **12 (4, 20)** | **17 (6, 26)** | **0.0004** |
| **Lesion characteristics** |  |  |  | |  |  |  |  |
| Number of diseased vessels | 1.9 ± 0.8 | 1.9 ± 0.8 | 0.84 | |  | 1.9 ± 0.8 | 2.1 ± 0.8 | 0.13 |
| Diseased Vessel |  |  |  | |  |  |  |  |
| RCA, n (%) | **49 (35.5)** | **42 (22.6)** | **0.01** | |  | 69 (30.0) | 108 (32.6) | 0.51 |
| LAD, n (%) | 68 (49.3) | 108 (58.1) | 0.12 | |  | 119 (51.7) | 154 (46.5) | 0.22 |
| LCX, n (%) | 22 (15.9) | 41 (22.0) | 0.17 | |  | 41 (17.8) | 65 (19.6) | 0.59 |
| **Laboratory findings** |  |  |  | |  |  |  |  |
| TC, mg/dL | 161.7 ± 33.1 | 160.4 ± 33.3 | 0.74 | |  | **174.8 ± 43.4** | **166.2 ± 38.7** | **0.01** |
| LDL-C, mg/dL (Friedewald) | 93.3 ± 26.5 | 92.2 ± 27.8 | 0.72 | |  | 100.9 ± 32.8 | 95.8 ± 30.2 | 0.06 |
| HDL-C, mg/dL | **40.2 ± 9.9** | **44.3 ± 13.9** | **0.003** | |  | **41.9 ± 11.9** | **45.1 ± 14.8** | **0.006** |
| TG, mg/dL | **127 (102, 173)** | **107 (79, 145)** | **0.0004** | |  | **134 (97, 187)** | **107 (81, 149)** | **<0.0001** |
| FBG, mg/dL | 134.2 ± 54.1 | 131.9 ± 52.5 | 0.71 | |  | 138.1 ± 56.1 | 134.7 ± 59.0 | 0.48 |
| HbA1c-NG, % | 7.1 ± 0.9 | 7.1 ± 1.0 | 0.94 | |  | **7.4 ± 1.3** | **7.2 ± 1.0** | **0.008** |
| hs-CRP, mg/L | 0.09 (0.04, 0.33) | 0.08 (0.03, 0.29) | 0.29 | |  | **0.12 (0.04, 0.37)** | **0.08 (0.03, 0.21)** | **0.02** |
| Hemoglobin, g/dL | **13.7 ± 1.8** | **13.1 ± 1.8** | **0.001** | |  | 13.6 ± 1.9 | 13.0 ± 2.0 | 0.006 |
| eGFR, ml/min/1.73m^2^ | 71.0 ± 27.3 | 71.3 ± 31.5 | 0.94 | |  | 69.2 ± 28.1 | 67.0 ± 27.5 | 0.36 |
| 1,5-AG, μg/mL | 9.1 (3.5, 15.3) | 8.4 (4.4, 14.0) | | 0.73 |  | 9.2 (4.5, 15.3) | 8.1 (4, 14.1) | 0.18 |
| BNP, pg/ml | 34.7 (14.8, 99.8) | 48.3 (25.1, 123.5) | 0.07 | |  | **43.3 (17.6, 118.1)** | **65.8 (28.7, 165.7)** | **0.001** |
| **Medications** |  |  |  | |  |  |  |  |
| Sulfonylurea, n (%) | 46 (33.3) | 53 (28.5) | 0.35 | |  | 54 (23.5) | 81 (24.5) | 0.79 |
| Metformin, n (%) | **45 (32.6)** | **32 (17.2)** | **0.001** | |  | 43 (18.7) | 50 (15.1) | 0.26 |
| Thiazolidinedione, n (%) | 16 (11.6) | 14 (7.5) | 0.21 | |  | **35 (15.2)** | **29 (8.8)** | **0.02** |
| SGLT-2 inhibitor, n (%) | **11 (8.0)** | **1 (0.5)** | **0.0005** | |  | 2 (0.9) | 3 (0.9) | 0.96 |
| GLP-1 receptor agonist, n (%) | 0 (0) | 1 (0.5) | 0.39 | |  | 4 (1.7) | 1 (0.3) | 0.07 |
| α-Glucosidase inhibitor, n (%) | 24 (17.4) | 38 (20.4) | 0.49 | |  | 44 (19.1) | 84 (25.4) | 0.08 |
| Glinide, n (%) | 11 (8.0) | 27 (14.5) | 0.07 | |  | **8 (3.5)** | **26 (7.9)** | **0.03** |
| Insulin, n (%) | 16 (11.6) | 32 (17.2) | 0.16 | |  | 69 (30.0) | 106 (32.0) | 0.61 |
| ACE-I/ ARB, n (%) | **87 (63.0)** | **85 (45.7)** | **0.002** | |  | 132 (57.4) | 181 (54.7) | 0.53 |
| β-Blocker, n (%) | 70 (50.7) | 79 (42.5) | 0.14 | |  | **121 (52.6)** | **135 (40.8)** | **0.006** |
| Statin, n (%) | 110 (79.7) | 132 (71.0) | 0.07 | |  | 142 (62.0) | 214 (64.7) | 0.52 |
| Ezetimibe, n (%) | 14 (10.1) | 12 (6.5) | 0.23 | |  | 11 (4.8) | 14 (4.2) | 0.75 |
| Fibrate, n (%) | **8 (5.8)** | **3 (1.6)** | **0.04** | |  | **15 (6.5)** | **8 (2.4)** | **0.02** |

**Supplementary Table 2**

Univariate Cox proportional hazard analyses for predictors of CV-death following PCI

|  | **HR** | **95% CI** | **p-value** |
| --- | --- | --- | --- |
| **Age (1 year older)** | 1.06 | 1.03–1.10 | **<0.0001** |
| Male | 1.71 | 0.83–4.12 | 0.184 |
| **BMI^1^ (1 kg/m^2^ higher)** | 0.87 | 0.80–0.94 | **0.001** |
| Hypertension | 1.01 | 0.54–2.04 | 0.986 |
| Dyslipidemia | 1.30 | 0.69–2.73 | 0.45 |
| Acute coronary syndrome | 1.08 | 0.59–1.88 | 0.795 |
| Multivessel disease | 1.20 | 0.69–2.16 | 0.54 |
| **Chronic kidney disease** | 2.61 | 1.56–4.36 | **0.0002** |
| **LVEF^2^ (1% higher)** | 0.96 | 0.94–0.98 | **0.0004** |
| **Former smoker** | 1.70 | 1.02–2.86 | **0.04** |
| Beta-blockers | 1.17 | 0.70–1.95 | 0.55 |
| ACEIs^3^/ARBs^4^ | 1.16 | 0.69–1.99 | 0.57 |
| Statins | 0.78 | 0.47–1.34 | 0.36 |
| Ezetimibe | 0.65 | 0.11–2.08 | 0.55 |
| **Hemoglobin (1 g/dL higher)** | 0.67 | 0.57–0.79 | **<0.0001** |
| **Triglycerides (1 mg/dL higher)** | 0.99 | 0.986–0.996 | **0.001** |
| **LDL-C^5^ (1 mg/dL higher)** | 0.99 | 0.979–0.999 | **0.04** |
| HDL-C^6^ (1 mg/dL higher) | 1.01 | 0.987–1.02 | 0.45 |
| **HbA1c-NG^7^ (1 % higher)** | 0.73 | 0.54–0.96 | **0.036** |
| **1,5-AG^8^ (1** μ**g/mL higher)** | 0.90 | 0.84–0.97 | **0.006** |
| IRI^9^ (1 μU/mL higher) | 1.00 | 0.98–1.02 | 0.67 |
| Fasting blood glucose (1 mg/dL higher) | 1.00 | 0.997–1.005 | 0.38 |
| **Log BNP^10^ (1 higher)** | 4.57 | 2.95–7.04 | **<0.0001** |
| **eGFR^11^ (10 mL/min/1.73m^2^ higher)** | 0.77 | 0.71–0.84 | **<0.0001** |
| **hs-CRP^12^ (0.1 mg/dL higher)** | 1.02 | 1.01–1.03 | **<0.0001** |
| **IGF-1^13^ (1 ng/mL higher)** | 0.99 | 0.979–0.997 | **0.009** |
| **IGFBP-3^14^ (1 ng/mL higher)** | 1.00 | 1.001–1.003 | **0.001** |
| **IGF-1/IGFBP-3 (quartile higher)** | 0.47 | 0.34–0.64 | **<0.0001** |
| Sulfonylurea | 0.60 | 0.30–1.10 | 0.12 |
| **Metformin** | 0.46 | 0.178–0.996 | **0.049** |
| Thiazolidinedione | 1.17 | 0.54–2.27 | 0.66 |
| α-Glucosidase inhibitor | 1.13 | 0.62–1.97 | 0.67 |
| Glinide | 0.59 | 0.14–1.58 | 0.37 |
| **Insulin** | 2.46 | 1.45–4.12 | **0.0007** |
| **DPP4i^15^** | 0.39 | 0.17–0.78 | **0.01** |
| 1 body mass index, 2 left ventricular ejection fraction, 3 angiotensin converting enzyme inhibitors, 4 angiotensin Ⅱ receptor blockers, 5 low-density lipoprotein, 6 high-density lipoprotein, 7 glycated hemoglobin, 8 1,5-anhydroglucitol, 9 immunoreactive insulin, 10 B-type natriuretic peptide, 11 estimated glomerular filtration rate, 12 high-sensitivity C-reactive protein, 13 insulin-like growth factor-1, 14 insulin-like growth factor binding protein-3, 15 dipeptidyl peptidase-4 inhibitors. | | | |

**Supplementary Table 3**

Univariate Cox proportional hazard analyses for predictors of 3P-MACE following PCI

|  | **HR** | **95% CI** | **p-value** |
| --- | --- | --- | --- |
| **Age (1 year older)** | 1.04 | 1.02–1.06 | **0.001** |
| Male | 1.47 | 0.83–2.84 | 0.19 |
| **BMI^1^ (1 kg/m^2^ higher)** | 0.93 | 0.87–0.99 | **0.03** |
| Hypertension | 1.17 | 0.69–2.11 | 0.58 |
| Dyslipidemia | 1.22 | 0.73–2.16 | 0.47 |
| Acute coronary syndrome | 1.23 | 0.76–1.91 | 0.39 |
| Multivessel disease | 1.33 | 0.84–2.17 | 0.23 |
| **Chronic kidney disease** | 2.34 | 1.54–3.55 | **<0.0001** |
| **LVEF^2^ (1% higher)** | 0.97 | 0.96–0.99 | **0.005** |
| Former smoker | 1.45 | 0.96–2.21 | 0.08 |
| Beta-blockers | 1.02 | 0.67–1.54 | 0.93 |
| ACEIs^3^/ARBs^4^ | 1.10 | 0.73–1.70 | 0.65 |
| Statins | 0.67 | 0.44–1.03 | 0.06 |
| Ezetimibe | 0.63 | 0.15–1.67 | 0.39 |
| **Hemoglobin (1 g/dL higher)** | 0.69 | 0.61–0.79 | **<0.0001** |
| **Triglycerides (1 mg/dL higher)** | 0.99 | 0.992–0.999 | **0.04** |
| LDL-C^5^ (1 mg/dL higher) | 0.99 | 0.988–1.002 | 0.29 |
| HDL-C^6^ (1 mg/dL higher) | 1.00 | 0.984–1.02 | 0.92 |
| HbA1c-NG^7^ (1 % higher) | 1.05 | 0.86–1.25 | 0.60 |
| **1,5-AG^8^ (1** μ**g/mL higher)** | 0.93 | 0.88–0.98 | **0.01** |
| IRI^9^ (1 μU/mL higher) | 1.01 | 0.99–1.02 | 0.25 |
| Fasting blood glucose (1 mg/dL higher) | 1.00 | 0.999–1.005 | 0.06 |
| **Log BNP^10^ (1 higher)** | 3.69 | 2.59–5.23 | **<0.0001** |
| **eGFR^11^ (10 mL/min/1.73m^2^ higher)** | 0.98 | 0.97–0.99 | **<0.0001** |
| **hs-CRP^12^ (0.1 mg/dL higher)** | 1.02 | 1.01–1.03 | **<0.0001** |
| **IGF-1^13^ (1 ng/mL higher)** | 0.99 | 0.985–0.999 | **0.04** |
| **IGFBP-3^14^ (1 ng/mL higher)** | 1.00 | 1.001–1.003 | **<0.0001** |
| **IGF-1/IGFBP-3 (quartile higher)** | 0.55 | 0.43–0.68 | **<0.0001** |
| Sulfonylurea | 0.64 | 0.37–1.04 | 0.07 |
| Metformin | 0.69 | 0.37–1.20 | 0.20 |
| Thiazolidinedione | 0.92 | 0.46–1.66 | 0.80 |
| α-Glucosidase inhibitor | 1.01 | 0.61–1.61 | 0.97 |
| Glinide | 0.97 | 0.41–1.94 | 0.93 |
| **Insulin** | 2.35 | 1.52–3.58 | **0.0002** |
| **DPP4i^15^** | 0.43 | 0.23–0.74 | **0.002** |
| 1 body mass index, 2 left ventricular ejection fraction, 3 angiotensin converting enzyme inhibitors, 4 angiotensin Ⅱ receptor blockers, 5 low-density lipoprotein, 6 high-density lipoprotein, 7 glycated hemoglobin, 8 1,5-anhydroglucitol, 9 immunoreactive insulin, 10 B-type natriuretic peptide, 11 estimated glomerular filtration rate, 12 high-sensitivity C-reactive protein, 13 insulin-like growth factor-1, 14 insulin-like growth factor binding protein-3, 15 dipeptidyl peptidase-4 inhibitors. | | | |

**Supplementary Table 4**

Multivariate cox proportional hazard analyses using 4 models assessed the hazard ratios of DPP4i for CV-death and 3P-MACE

**Model 1**: Hazard ratios for dipeptidyl peptidase-4 inhibitors (DPP4i) were adjusted by age and sex

**For CV-death**

|  | **HR** | **95% CI** | **p-value** |
| --- | --- | --- | --- |
| **Age (1 year older)** | **1.07** | **1.04–1.10** | **<0.0001** |
| Male | 2.04 | 0.99–4.94 | 0.054 |
| **DPP4i** | **0.38** | **0.16–0.75** | **0.005** |

**For 3P-MACE**

|  | **HR** | **95% CI** | **p-value** |
| --- | --- | --- | --- |
| **Age (1 year older)** | **1.04** | **1.02–1.07** | **0.0004** |
| Male | 1.67 | 0.94–3.23 | 0.08 |
| **DPP4i** | **0.42** | **0.23–0.73** | **0.001** |

**Model 2**: Hazard ratios for dipeptidyl peptidase-4 inhibitors (DPP4i) were adjusted by age, sex, body mass index (BMI), chronic kidney disease (CKD), BNP (B-type natriuretic peptide) and insulin use

**For CV-death**

|  | **HR** | **95% CI** | **p-value** |
| --- | --- | --- | --- |
| **Age (1 year older)** | **1.06** | **1.03–1.09** | **0.0003** |
| **Male** | **3.88** | **1.66–11.35** | **0.0009** |
| BMI (1 kg/m^2^ higher) | 0.93 | 0.85–1.02 | 0.14 |
| Chronic kidney disease | 0.98 | 0.53–1.81 | 0.95 |
| **Log BNP (1 higher)** | **5.02** | **2.96–8.52** | **<0.0001** |
| **Insulin** | **2.26** | **1.24–4.05** | **0.009** |
| **DPP4i** | **0.39** | **0.16–0.82** | **0.01** |

**For 3P-MACE**

|  | **HR** | **95% CI** | **p-value** |
| --- | --- | --- | --- |
| **Age (1 year older)** | **1.03** | **1.00–1.06** | **0.01** |
| **Male** | **2.45** | **1.31–5.13** | **0.004** |
| BMI (1 kg/m^2^ higher) | 0.99 | 0.93–1.06 | 0.81 |
| Chronic kidney disease | 1.17 | 0.71–1.91 | 0.54 |
| **Log BNP (1 higher)** | **3.61** | **2.40–5.44** | **<0.0001** |
| **Insulin** | **1.92** | **1.19–3.06** | **0.009** |
| **DPP4i** | **0.47** | **0.25–0.84** | **0.01** |

**Model 3:** Hazard ratios for dipeptidyl peptidase-4 inhibitors (DPP4i) were adjusted by age, sex, acute coronary syndrome (ACS), left ventricular ejection fraction (LVEF), statins and hemoglobin

**For CV-death**

|  | **HR** | **95% CI** | **p-value** |
| --- | --- | --- | --- |
| **Age (1 year older)** | **1.05** | **1.01–1.09** | **0.01** |
| **Male** | **4.28** | **1.50–18.05** | **0.004** |
| ACS | 0.74 | 0.29–1.73 | 0.50 |
| LVEF | 1.00 | 0.98–1.01 | 0.46 |
| statins | 0.82 | 0.42–1.70 | 0.59 |
| **Hemoglobin (1 g/dL higher)** | **0.66** | **0.55–0.78** | **<0.0001** |
| **DPP4i** | **0.44** | **0.19–0.95** | **0.04** |

**For 3P-MACE**

|  | **HR** | **95% CI** | **p-value** |
| --- | --- | --- | --- |
| **Age (1 year older)** | **1.03** | **1.00–1.06** | **0.04** |
| **Male** | **3.35** | **1.44–9.75** | **0.003** |
| ACS | 0.98 | 0.46–1.94 | 0.95 |
| LVEF | 1.00 | 0.99–1.01 | 0.91 |
| statins | 0.79 | 0.44–1.43 | 0.42 |
| **Hemoglobin (1 g/dL higher)** | **0.67** | **0.58–0.77** | **<0.0001** |
| DPP4i | 0.55 | 0.29–1.01 | 0.052 |

**Model 4:** Adjusted by anti-diabetic medications

**For CV-death**

|  | **HR** | **95% CI** | **p-value** |
| --- | --- | --- | --- |
| Metformin | 0.50 | 0.19–1.10 | 0.12 |
| Sulfonylurea | 0.72 | 0.35–1.39 | 0.33 |
| Alpha-glucosidase inhibitors | 1.23 | 0.66–2.18 | 0.50 |
| Thiazolidinedione | 1.40 | 0.63–2.76 | 0.38 |
| Glinide | 0.64 | 0.15–1.78 | 0.43 |
| **Insulin** | **2.11** | **1.21–3.62** | **0.009** |
| **DPP4i** | **0.48** | **0.21–0.99** | **0.046** |

**For 3P-MACE**

|  | **HR** | **95% CI** | **p-value** |
| --- | --- | --- | --- |
| Metformin | 0.75 | 0.39–1.32 | 0.33 |
| Sulfonylurea | 0.79 | 0.44–1.35 | 0.40 |
| Alpha-glucosidase inhibitors | 1.06 | 0.63–1.71 | 0.82 |
| Thiazolidinedione | 1.03 | 0.51–1.87 | 0.94 |
| Glinide | 1.08 | 0.44–2.22 | 0.86 |
| **Insulin** | **2.05** | **1.30–3.18** | **0.002** |
| **DPP4i** | **0.51** | **0.27–0.88** | **0.02** |

**Supplementary Table 5**

Multivariate Cox proportional hazard analyses using Model 2 assessed the hazard ratios of IGF-1/IGFBP-3 ratio for CV-death and 3P-MACE

**Model 2**: Hazard ratios for dipeptidyl peptidase-4 inhibitors (DPP4i) were adjusted by age, sex, body mass index (BMI), chronic kidney disease (CKD), BNP (B-type natriuretic peptide) and insulin use

**For CV-death**

|  | **HR** | **95% CI** | **p-value** |
| --- | --- | --- | --- |
| **Age (1 year older)** | 1.05 | 1.01–1.09 | **0.01** |
| **Male** | 4.62 | 1.77–15.83 | **0.0009** |
| BMI (1 kg/m^2^ higher) | 0.96 | 0.87–1.06 | 0.47 |
| Chronic kidney disease | 0.83 | 0.39–1.75 | 0.63 |
| **Log BNP (1 higher)** | 3.32 | 1.83–6.07 | **<0.0001** |
| **Insulin** | 2.83 | 1.38–5.69 | **0.005** |
| **IGF-1/IGFBP-3 (quartile increase)** | 0.58 | 0.40–0.80 | **0.002** |

**For 3P-MACE**

|  | **HR** | **95% CI** | **p-value** |
| --- | --- | --- | --- |
| Age (1 year older) | 1.01 | 0.99–1.05 | 0.31 |
| **Male** | 2.99 | 1.46–6.96 | **0.002** |
| BMI (1 kg/m^2^ higher) | 0.99 | 0.92–1.07 | 0.89 |
| Chronic kidney disease | 1.03 | 0.56–1.86 | 0.93 |
| **Log BNP (1 higher)** | 2.70 | 1.69–4.30 | **<0.0001** |
| **Insulin** | 2.31 | 1.31–4.01 | **0.004** |
| **IGF-1/IGFBP-3 (quartile increase)** | 0.59 | 0.45–0.77 | **0.0001** |


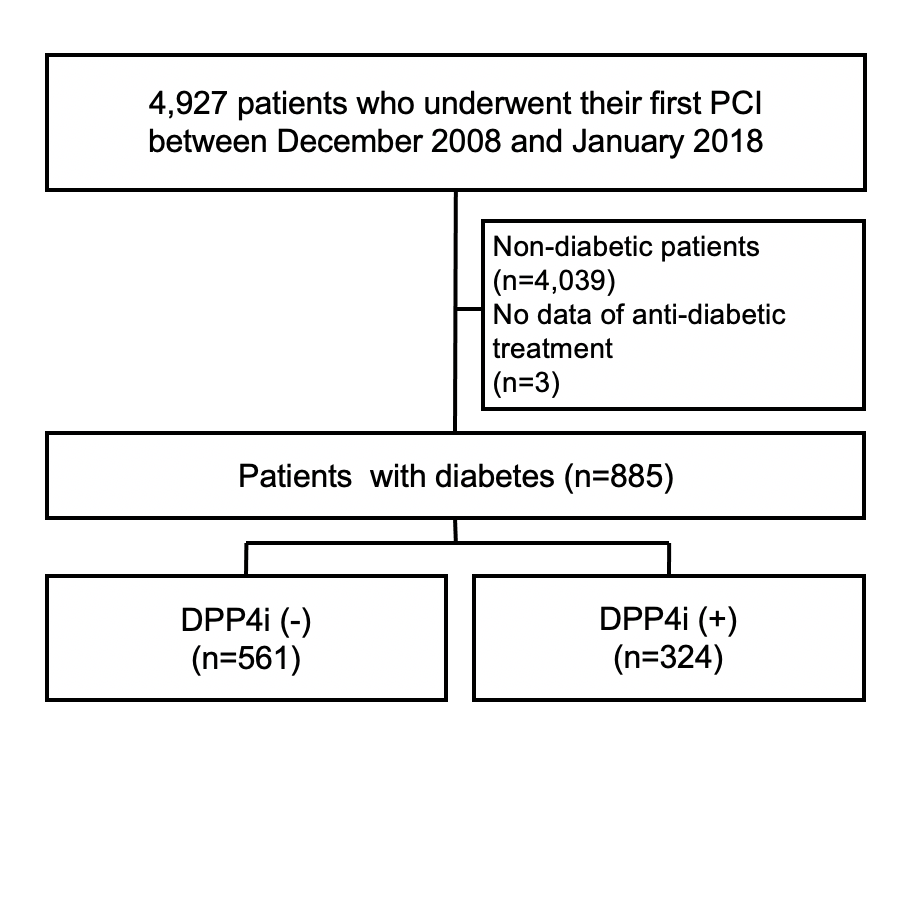
**Supplementary Figure 1**

**Supplementary Figure 1: Consort diagram**

**Supplementary Figure 2**

**
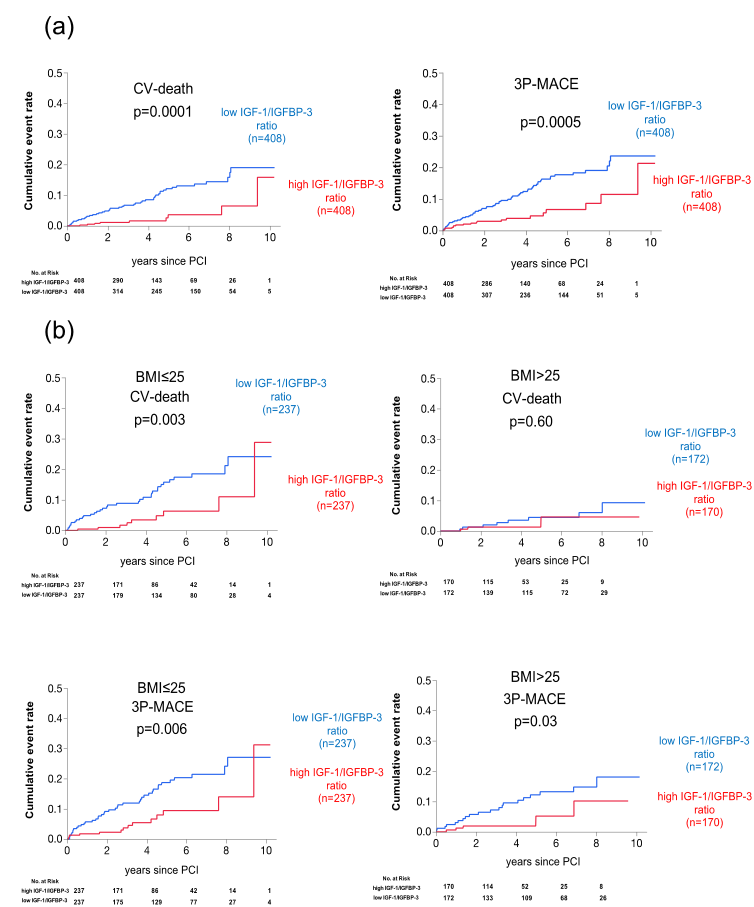
**

**Supplementary Figure 2: Cumulative incidence of adverse outcomes following PCI in two groups divided by the median of IGF-1/IGFBP-3 ratio**

(a) Cumulative incidences of cardiovascular (CV)-death and the composite of CV-death, non-fatal myocardial infarction and ischemic stroke (3P-MACE) in the high and low IGF-1/IGFBP-3 ratio groups. (b) Cumulative incidences of CV-death and 3P-MACE in the high and low IGF-1/IGFBP-3 ratio groups in patients with and without low BMI (≤ and > median BMI, 25).
